# Computed Tomography-Based Radiomics Provides New Insights Into Associations Between Pericoronary Fat Characteristics and Low-Density Lipoprotein Cholesterol

**DOI:** 10.31083/RCM47037

**Published:** 2026-05-25

**Authors:** Feifei Zhou, Xingrui Liu, Lei Yang, Yang Zhang, Yongju Yang, Shiying Tang, Xinyan Zhou, Xirui Duan, Na Tan, Shuaiyan Zuo, Fei Liu, Yan Xu, Caiyan Zhu, Lishi Shao, Guifang Sun

**Affiliations:** ^1^Department of Radiology, Kunming Yan’an Hospital (Yan’an Hospital Affiliated to Kunming Medical University), 650051 Kunming, Yunnan, China; ^2^Department of Vascular Surgery, Fuwai Yunnan Hospital Chinese Academy of Medical Sciences, 650032 Kunming, Yunnan, China

**Keywords:** radiomics, computed tomography angiography, epicardial adipose tissue, cholesterol, LDL, coronary artery disease

## Abstract

**Background:**

Pericoronary adipose tissue (PCAT) is an established imaging biomarker of coronary inflammation; however, the influence of low-density lipoprotein cholesterol (LDL-C) remains unclear. This study aimed to explore the associations between PCAT and LDL-C using coronary computed tomography angiography (CCTA)-based radiomics.

**Methods:**

This retrospective study stratified 150 patients undergoing CCTA into two groups according to serum LDL-C levels (≥3.4 mmol/L vs. <3.4 mmol/L). A total of 288 radiomic features were extracted from the PCAT surrounding the left anterior descending artery, left circumflex artery, and right coronary artery. After the initial filtering using the Wilcoxon rank-sum test, univariate logistic regression and Pearson correlation analyses were applied to identify features associated with elevated LDL-C levels. Key features were further validated using a gradient boosting machine (GBM) ensemble model combined with Shapley Additive Explanations (SHAP) analysis.

**Results:**

A total of 11 radiomic features were significantly associated with elevated LDL-C levels (*p* < 0.05), including both first-order and texture-based features. Mantel correlation analysis revealed that the gray level size zone matrix (GLSZM)-derived feature, GLSZM.LCXLargeAreaHighGrayLevelEmphasis, demonstrated the strongest association (Mantel's r ≈ 0.15; *p* < 0.01). The GBM model achieved the best performance, with an area under the receiver operating characteristic curve (AUROC) of 0.889 in the training set and 0.724 in an internal hold-out test set. SHAP analysis identified first-order energy and large-area high gray-level features as the most important contributors to the discrimination of high LDL-C status.

**Conclusion:**

Elevated LDL-C levels are significantly associated with increased spatial heterogeneity and high gray-level clustering in PCAT, thereby providing imaging-based evidence supporting the association between LDL-C and PCAT.

## 1. Introduction

In the routine evaluation of atherosclerosis, patients typically undergo both lipid profiling, including measurement of low-density lipoprotein cholesterol (LDL-C), and coronary computed tomography angiography (CCTA). Clinically, elevated LDL-C is often accompanied by CCTA abnormalities such as non-calcified plaques, high-risk plaques, and changes in pericoronary adipose tissue (PCAT) attenuation [1, 2, 3]. In some patients, LDL-C elevation coincides with reduced PCAT attenuation, which may represent different stages of the same pathological process. However, the patient-level relationship between LDL-C and PCAT phenotype has not been systematically quantified, and existing evidence has rarely moved beyond qualitative observations or single-parameter imaging summaries.

Evidence-based research has established low-density lipoprotein (LDL) as a causal factor in atherosclerotic cardiovascular disease (ASCVD) [3, 4] and has demonstrated a dose-dependent, log-linear relationship between cumulative LDL-C exposure and cardiovascular event risk, with longer exposure associated with greater risk [5, 6]. Recent clinical evidence further suggests that statin therapy is associated with a reduction in PCAT lesion attenuation, whereas no significant changes are observed in the absence of statin treatment [7, 8]. After adjustment for cardiovascular risk factors, changes in LDL-C are independently associated with the percentage change in PCAT lesions. In a cohort of 180 patients with chest pain and intermediate risk of coronary artery disease [9], ≥1 year of statin therapy reduced the mean CT attenuation of perivascular adipose tissue (PVAT) surrounding non-calcified plaques from –68 ± 9 Hounsfield unit (HU) to –72 ± 8 HU (*p* < 0.001), whereas no significant change was observed in PVAT attenuation surrounding calcified plaques. These findings suggest that the lipid status surrounding the coronary arteries may be reflected in the local PCAT phenotype in an imageable manner; however, existing evidence largely remains focused on the single imaging parameter of mean PCAT attenuation.

Whether LDL-C is associated with more complex PCAT microstructural signatures (e.g., spatial heterogeneity and texture patterns) on CCTA remains unclear.

Although previous studies have established PCAT as an imaging biomarker of coronary inflammation [10, 11], capable of improving coronary heart disease risk stratification and independently associated with adverse cardiovascular events [12, 13, 14], a single HU value alone cannot fully capture the complex alterations in adipose tissue microstructure and metabolic inflammation. Radiomics, through high-precision segmentation and computational algorithms [15, 16], can automatically extract multidimensional features from CCTA images, including first-order statistics, texture, and higher-order filtered features, thereby capturing tissue characteristics at multiple spatial scales [17]. Applying radiomics to PCAT enables in-depth characterization of its microstructural patterns, thereby overcoming the limitations of reliance on a single HU value [18].

Despite these observations, key gaps remain: the patient-level association between LDL-C and PCAT has rarely been quantified beyond mean attenuation, and informative PCAT radiomic signatures related to elevated LDL-C have not been systematically evaluated. To address these gaps, we (1) quantified multidimensional PCAT radiomic features on routine CCTA; (2) identified key features associated with elevated LDL-C using an internal training/hold-out evaluation strategy; and (3) applied Shapley additive explanations (SHAP)-based interpretation to improve model interpretability.

Based on these considerations, the aim of this study was to extract and select PCAT radiomic features from CCTA images of patients with varying lipid levels and to systematically assess their correlation with elevated LDL-C levels, identify key features most closely associated with LDL-C levels, and explore their potential value in elucidating the mechanisms of LDL-C–related coronary inflammation, thereby providing evidence to support the precise clinical evaluation and management of LDL-C–related cardiovascular risk.

## 2. Methods

### 2.1 Study Design and Patient Population

This retrospective study included 150 adult patients (≥18 years) who underwent clinically indicated CCTA between May 2023 and March 2024. Consecutive eligible patients were identified from the institutional CCTA database (Picture Archiving and Communication System/Radiology Information System [PACS/RIS]) of Kunming Yan’an Hospital during the study period. The inclusion criteria were as follows: (1) CCTA of adequate image quality enabling complete segmentation of PCAT surrounding the left anterior descending artery (LAD), left circumflex artery (LCX), and right coronary artery (RCA); and (2) availability of serum LDL-C measurements obtained on the day of CCTA. The exclusion criteria were incomplete clinical data, impaired cardiac or renal function, autoimmune diseases, or hematologic disorders. Patients were stratified into elevated LDL-C levels (n = 74) and lower LDL-C levels (n = 76) groups using an LDL-C threshold of 3.4 mmol/L (≈130 mg/dL), which corresponds to the lower boundary of the “borderline-high” LDL-C category in the National Cholesterol Education Program Adult Treatment Panel III (NCEP ATP III) classification [19]. Patients receiving lipid-lowering therapy (e.g., statins) were not excluded and treated and untreated individuals were included to reflect routine clinical practice. The study protocol complied with all relevant regulations and institutional guidelines and was approved by the Ethics Committee of Kunming Yan’an Hospital (Kunming, China; Approval No. 2023-083-01). Written informed consent was not required.

### 2.2 CCTA Image Acquisition

CCTA examinations were performed using a dual-source system (SOMATOM Definition Flash; Siemens Healthineers, Forchheim, Germany). Depending on patient condition and heart rate, data were obtained using prospective electrocardiography (ECG)-triggered or retrospective ECG-gated techniques. Immediately before image acquisition, sublingual nitroglycerin (0.5 mg) was administered to all participants. Contrast enhancement was achieved using iodixanol (320 mg I/mL; No. H20113465, Beijing Beilu Pharmaceutical Co., Ltd., Beijing, China), delivered with a dual-head injector (Bayer; Stellant D-CE, REF: 84723223, Leverkusen, Germany) in three phases: 60 mL at 5 mL/s, then 30 mL of a 30% contrast/70% saline mixture, then a 30 mL saline chaser. Bolus tracking was performed in the ascending aorta, and scanning was triggered 4 s after peak enhancement. Typical parameters included a tube potential of 80–100 kVp, an effective tube current of 500 mA, a reconstructed slice thickness of 0.5 mm, and a gantry rotation time of 0.28 s. Images were reconstructed using a standard cardiac kernel and processed on a dedicated workstation (syngo.via, version 8.13; Siemens Healthineers, Forchheim, Germany) for multiplanar reformation and maximum intensity projection. All images were acquired at a single center using the same scanner and following a standardized acquisition and reconstruction protocol.

### 2.3 CCTA Image Quality Assessment

All CCTA images were independently evaluated by two radiologists with 10 years of clinical experience, using a 4-point Likert scoring system [20]: 1 point (poor): severe image noise with blurred vascular borders, rendering the images unsuitable for analysis; 2 points (fair): noticeable noise but acceptable contrast and resolution, with identifiable vascular contours; 3 points (good): minimal noise interference, high contrast and resolution, with clear vascular margins; 4 points (excellent): no image noise, sharply defined vessel walls, and excellent image resolution. After independent scoring, any discrepancies were resolved by consensus through joint review. Images rated as 1 point were excluded because of suboptimal quality. Only CCTA images with a score of ≥2 were included in the study to ensure the accuracy of subsequent PCAT segmentation and radiomics analysis.

### 2.4 PCAT Radiomics Feature Extraction and Selection

**Fig. 1.**
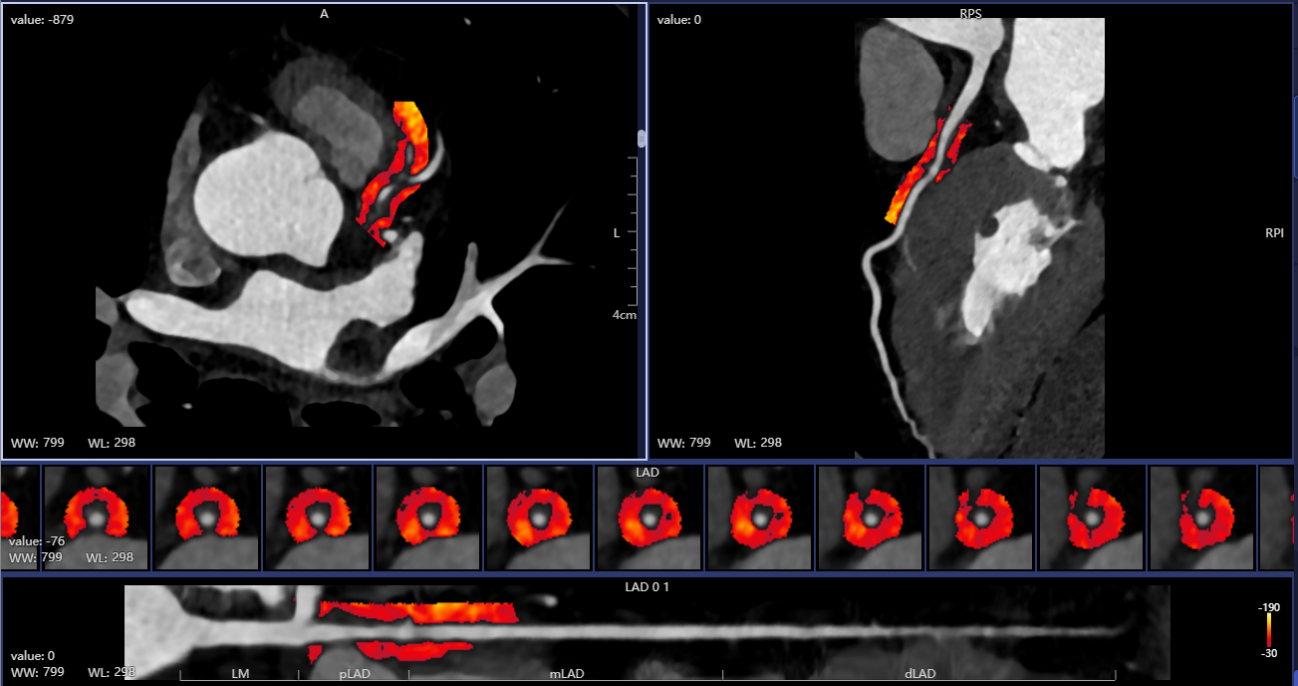
**Automatic drawing of pericoronary adipose tissue (PCAT)**. LAD, left anterior descending artery; WW, window width; WL, window level; LM, left main; pLAD, proximal Left Anterior Descending; mLAD, mid Left Anterior Descending; dLAD, distal Left Anterior Descending.

PCAT was delineated as voxels located within 3 mm of the external coronary vessel boundary and restricted to attenuation values between –190 and –30 HU [10]. The regions of interest (ROIs) were segmented along the proximal 10–50 mm of the main coronary arteries, including the LAD, LCX, and RCA [21, 22]. All PCAT segmentations and voxel extractions were automatically performed using an AI-assisted software platform (CoronaryDoc, version 1.0; Shukun Technology Co., Ltd., Beijing, China), which employed deep learning algorithms to identify perivascular fat and extract voxel data from predefined ROIs (Fig. 1).

Radiomic features were extracted from each ROI using PyRadiomics (version 3.0; open-source Python package, Boston, MA, USA) embedded within the CoronaryDoc platform. A total of 288 features were obtained, including first-order statistics, shape-based features, gray-level co-occurrence matrix (GLCM), gray-level size zone matrix (GLSZM), gray-level run-length matrix (GLRLM), neighboring gray-tone difference matrix (NGTDM), and gray-level dependence matrix features.

To identify key imaging features closely associated with elevated LDL-C levels (≥3.4 mmol/L), a systematic feature selection process was performed as follows. As an initial filter, the Wilcoxon rank-sum test was used to screen features showing between-group differences; features passing this step were then evaluated using univariate logistic regression with false discovery rate (FDR) correction to identify variables associated with elevated LDL-C (*p* < 0.05). Univariate logistic regression was applied to the imaging features to identify variables significantly associated with elevated LDL-C levels (*p* < 0.05); these variables were then used as candidate features for machine learning. Twelve machine learning algorithms were combined in various pairs, including least absolute shrinkage and selection operator (LASSO), Ridge, elastic net (Enet), stepwise generalized linear model (Stepglm), support vector machine (SVM), glmBoost, linear discriminant analysis (LDA), partial least squares regression with generalized linear modeling (plsRglm), random forest, gradient boosting machine (GBM), extreme gradient boosting (XGBoost), and naïve bayes, In each pair, one algorithm was used for feature selection and the other for classification model construction within a cross-validation framework. The dataset was randomly split into a training set (70%) and an internal hold-out test set (30%). All model selection and tuning procedures were conducted within the training set only. Five-fold cross-validation was performed within the training set for model selection and tuning. The final selected model was evaluated in the internal hold-out test set. The GBM model was implemented using the R package gbm (version 2.2.2; Comprehensive R Archive Network [CRAN]). Model performance was quantified using the area under the receiver operating characteristic curve (AUC), reported for both the training and internal test sets. SHAP was used to interpret the contribution of the selected radiomic features.

### 2.5 Statistical Analysis

All analyses were conducted using R software (version 4.3.2; The R Foundation for Statistical Computing, Vienna, Austria). Data completeness was assessed prior to analysis, and no missing values were present in the final analytic dataset; therefore, no imputation was required. Continuous variables were assessed using independent-sample *t*-tests or Mann–Whitney U tests, whereas categorical variables were evaluated using χ^2^ or Fisher’s exact tests. For multiple testing, p values were corrected using the Benjamini–Hochberg procedure, with a false discovery rate <0.05 as considered statistically significant. Univariate logistic regression was used to assess the relationship between radiomic features and elevated LDL-C levels (≥3.4 mmol/L). Model interpretation was performed using SHAP through the R packages shapviz and fastshap. Correlations between the final selected features and clinical parameters, including total cholesterol (TC), high-density lipoprotein cholesterol (HDL-C), liver function indices, and inflammatory markers, were assessed using Pearson correlation analysis. The consistency between the radiomic feature space and LDL-C group stratification was evaluated using the Mantel test.

## 3. Results

### 3.1 Patient Characteristics

A total of 150 patients were included and were stratified into elevated LDL-C levels (n = 74) and lower LDL-C levels (n = 76). The sex distribution was comparable between the two groups (male, 54% vs. 57%, *p* = 0.80) and body mass index (BMI) was similar (24.01 (2.83)/23.41 (4.24) kg/m^2^, *p* = 0.074). Regarding lipid profiles, the elevated LDL-C levels group had higher TC (5.10 ± 0.87 vs. 3.92 ± 0.84 mmol/L, *p* < 0.001) and slightly higher HDL-C levels (1.18 ± 0.32 vs. 1.08 ± 0.29 mmol/L, *p* = 0.041). Liver-related indices were higher for alanine aminotransferase (ALT), total protein (TP), albumin (Alb), globulin (Glb), and cholinesterase (ChE), whereas total bile acid (TBA) was lower (all *p* ≤ 0.024). No significant differences were observed in systolic blood pressure (SBP), triglyceride (TG), high-sensitivity C-reactive protein (hs-CRP), smoking, drinking, or hypertension (all *p* ≥ 0.05). Further details are provided in Table 1.

**Table 1.** **Baseline characteristics of patients stratified by LDL-C levels**.

Characteristic	LDL-C <3.4 (n = 76)	LDL-C ≥3.4 (n = 74)	*p* value
Age (years)	62.3 ± 12.3	57.3 ± 11.6	0.018
Gender	Male: 43 (57%) Female: 33 (43%)	Male: 40 (54%) Female: 34 (46%)	0.80
BMI (kg/m^2^)	23.41 (4.24)	24.01 (2.83)	0.074
HR (bpm)	80.7 ± 15.5	74.6 ± 12.2	0.023
DBP (mmHg)	80.4 ± 13.7	87.6 ± 19.9	0.039
SBP (mmHg)	133.53 (25.46)	141.18 (31.54)	0.20
Hypertension	37 (49%)	43 (58%)	0.20
Drinking	1 (1.3%)	2 (2.7%)	0.60
Smoking	1 (1.3%)	3 (4.1%)	0.40
TC (mmol/L)	3.92 ± 0.84	5.10 ± 0.87	<0.001
TG (mmol/L)	2.16 ± 2.25	1.75 ± 0.68	0.20
HDL-C (mmol/L)	1.08 ± 0.29	1.18 ± 0.32	0.041
ALT (U/L)	23.3 ± 18.4	28.5 ± 21.6	0.018
TBA (µmol/L)	5.56 ± 6.14	3.40 ± 2.44	0.006
TP (g/L)	66.4 ± 5.9	69.7 ± 6.9	0.001
Alb (g/L)	39.2 ± 4.5	41.3 ± 4.0	0.001
Glb (g/L)	27.3 ± 3.5	28.4 ± 4.0	0.024
ChE (U/L)	6543 ± 1999	7440 ± 1553	0.002
hs-CRP (mg/L)	2.1 ± 1.8	2.3 ± 1.9	0.50

Values are mean ± SD, n (%), or median (interquartile range [IQR]). Abbreviations: Alb, albumin; ALT, alanine aminotransferase; BMI, body mass index; ChE, cholinesterase; DBP, diastolic blood pressure; Glb, globulin; HDL-C, high-density lipoprotein cholesterol; HR, heart rate; hs-CRP, high-sensitivity C-reactive protein; IQR, interquartile range; LDL-C, low-density lipoprotein cholesterol; SBP, systolic blood pressure; TBA, total bile acid; TC, total cholesterol; TG, triglyceride; TP, total protein.

### 3.2 PCAT Radiomics Feature Extraction and Selection

**Fig. 2.**
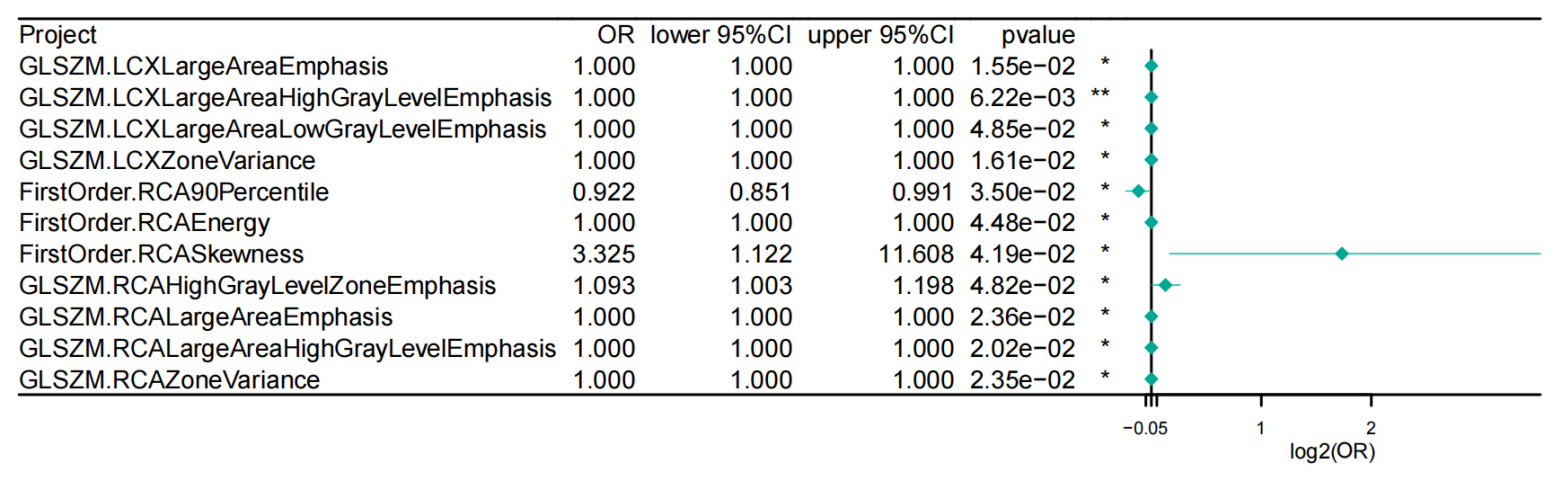
**Univariate logistic regression results of 11 selected PCAT radiomic features**. Abbreviations: CI, confidence interval; FirstOrder, first-order statistics (radiomic feature class); GLSZM, gray level size zone matrix; LCX, left circumflex artery; OR, odds ratio; PCAT, pericoronary adipose tissue; RCA, right coronary artery. Statistical significance: *p < 0.05, **p < 0.01.

A total of 288 radiomics features were initially extracted from the PCAT ROIs surrounding the LAD, LCX, and RCA. Preliminary screening using the Wilcoxon rank-sum test identified 48 candidate features showing statistically significant differences between the groups (**Supplementary Table 1**). These candidate features were then individually entered into univariate logistic regression analyses to assess their associations with elevated LDL-C levels (≥3.4 mmol/L), yielding 11 features that were retained as the core feature set for subsequent analyses (Fig. 2). No high collinearity was detected among these features (all |r| < 0.90), indicating independence within the feature set.

Finally, all 11 features were incorporated into hybrid ensemble models with embedded feature selection capability, including random forest with LASSO regularization combined with Elastic Net, and SVM combined with GBM (**Supplementary Fig. 1**), and were subjected to five-fold cross-validation within the training set to assess feature stability and importance. Among all algorithmic combinations, the GBM-based model demonstrated the best performance, and no further features were eliminated. Therefore, these 11 features were identified as the final PCAT radiomics feature set and were used for subsequent correlation analyses and SHAP-based interpretability assessment.

### 3.3 Correlation of Radiomic Features With Clinical Variables

**Fig. 3.**
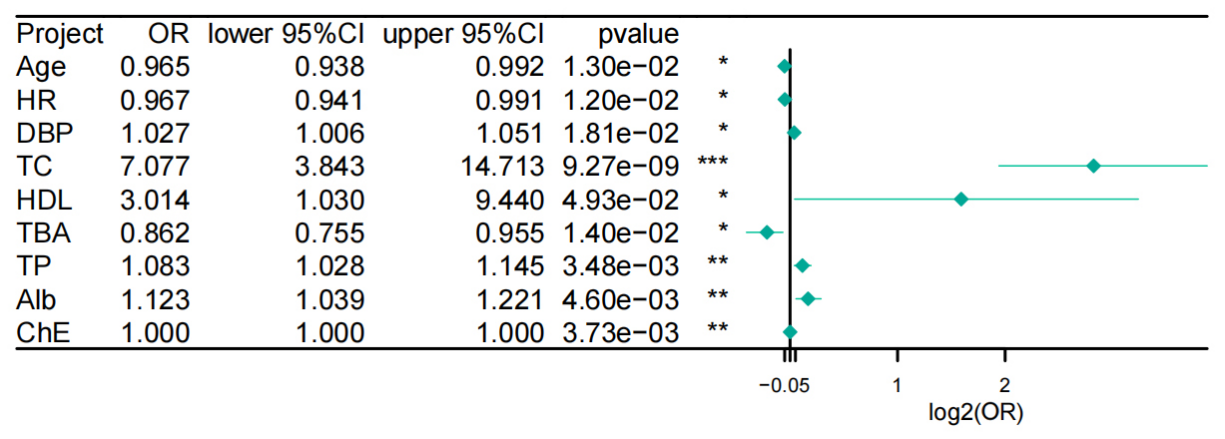
**Univariate logistic regression forest plot of clinical variables associated with elevated LDL-C levels**. Abbreviations: CI, confidence interval; HDL, high-density lipoprotein cholesterol; OR, odds ratio. Statistical significance: *p < 0.05, **p < 0.01, ***p < 0.001.

**Fig. 4.**
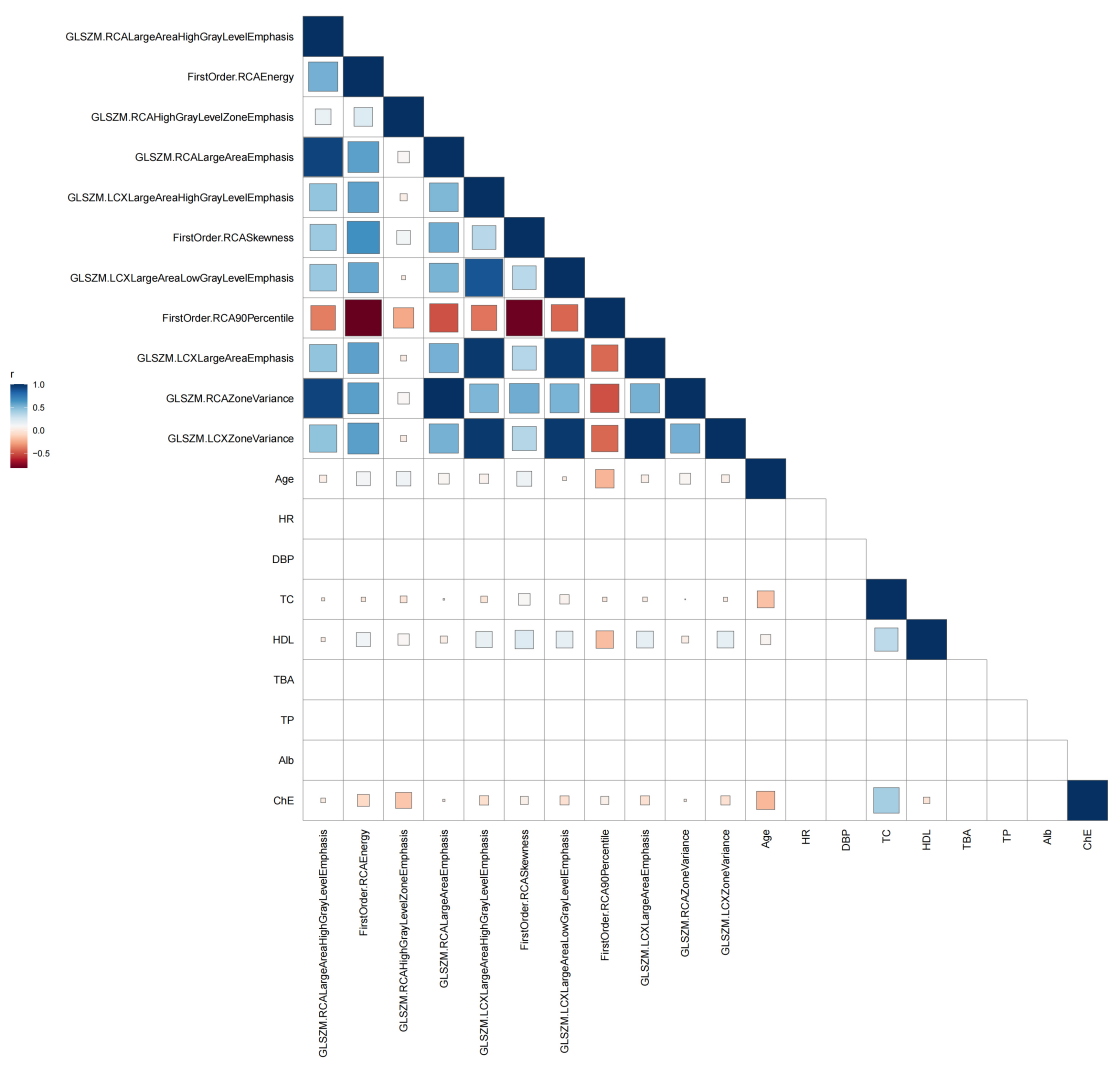
**Pearson correlation heatmap between PCAT radiomic features and clinical variables**. Abbreviations: HDL, high-density lipoprotein cholesterol; PCAT, pericoronary adipose tissue.

In the univariate logistic regression analysis of clinical variables, both TC and HDL-C were significantly associated with elevated LDL-C levels (Fig. 3). Based on the 11 PCAT radiomic features retained in Section 3.2, Pearson correlation analysis was performed with key clinical variables, including TC, HDL-C, and liver function parameters (Fig. 4). Overall, the correlations were predominantly weak to moderate; among them, HDL-C demonstrated positive correlations with several GLSZM and first-order features. Other clinical variables exhibited generally weak correlations with most imaging features (|r| < 0.2), and no consistent patterns were observed.

### 3.4 Association Between PCAT Radiomic Features and LDL-C

Mantel correlation analysis demonstrated a significant association between PCAT structural features and elevated LDL-C levels. Among the 11 evaluated texture features, four showed significant positive correlations with GLSZM.LCXLargeAreaHighGrayLevelEmphasis demonstrates the strongest association (Mantel’s r ≈ 0.15, *p* < 0.01). Additionally, GLSZM.LCXLargeAreaLowGrayLevelEmphasis, GLSZM.LCXLargeAreaEmphasis, and GLSZM.LCXZoneVariance also displayed moderate correlations (*p* < 0.05).

**Fig. 5.**
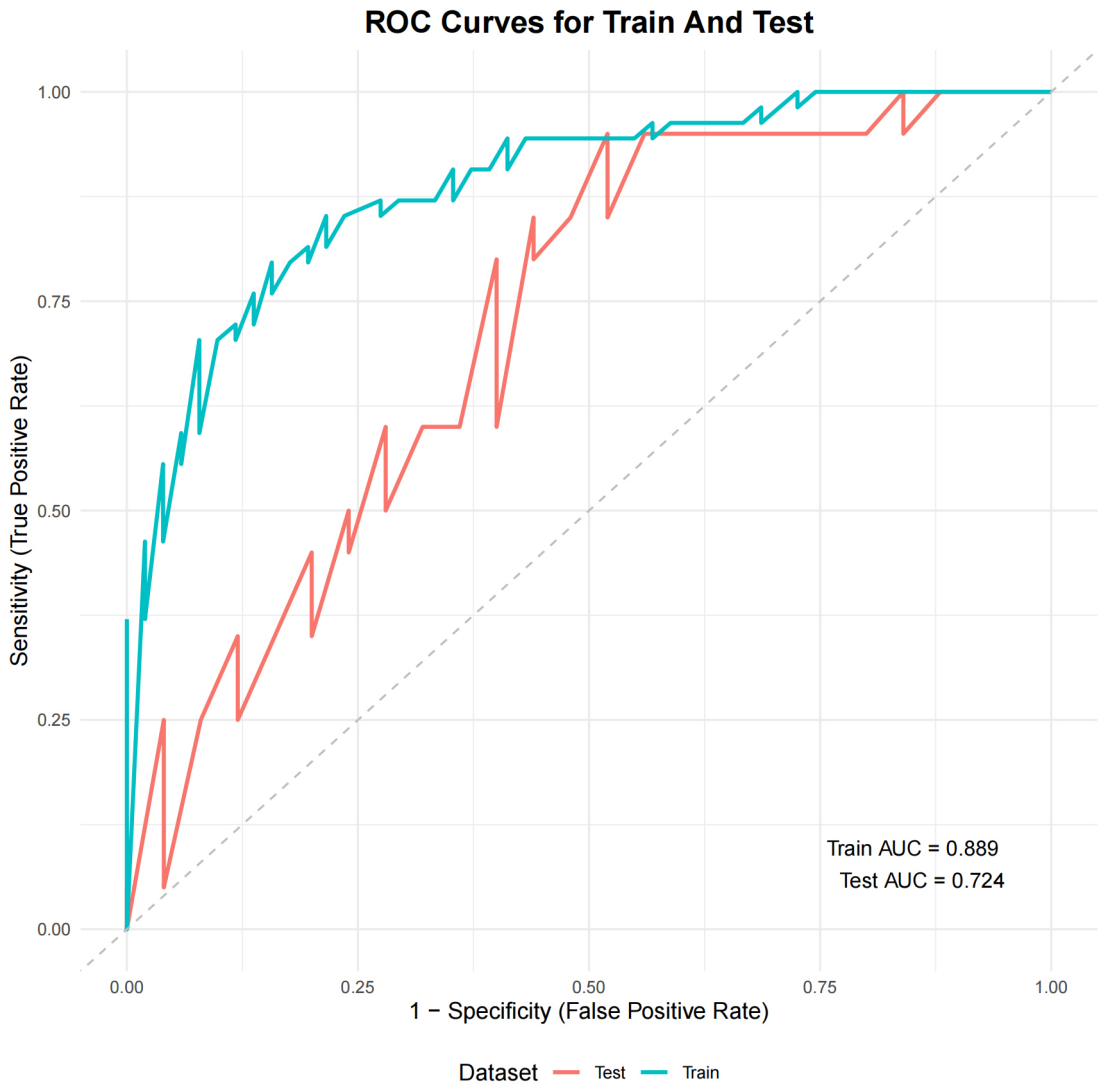
**ROC curves of the GBM model for training and test sets**. Abbreviations: AUC, area under the curve; GBM, gradient boosting machine; ROC, receiver-operating characteristic.

**Fig. 6.**
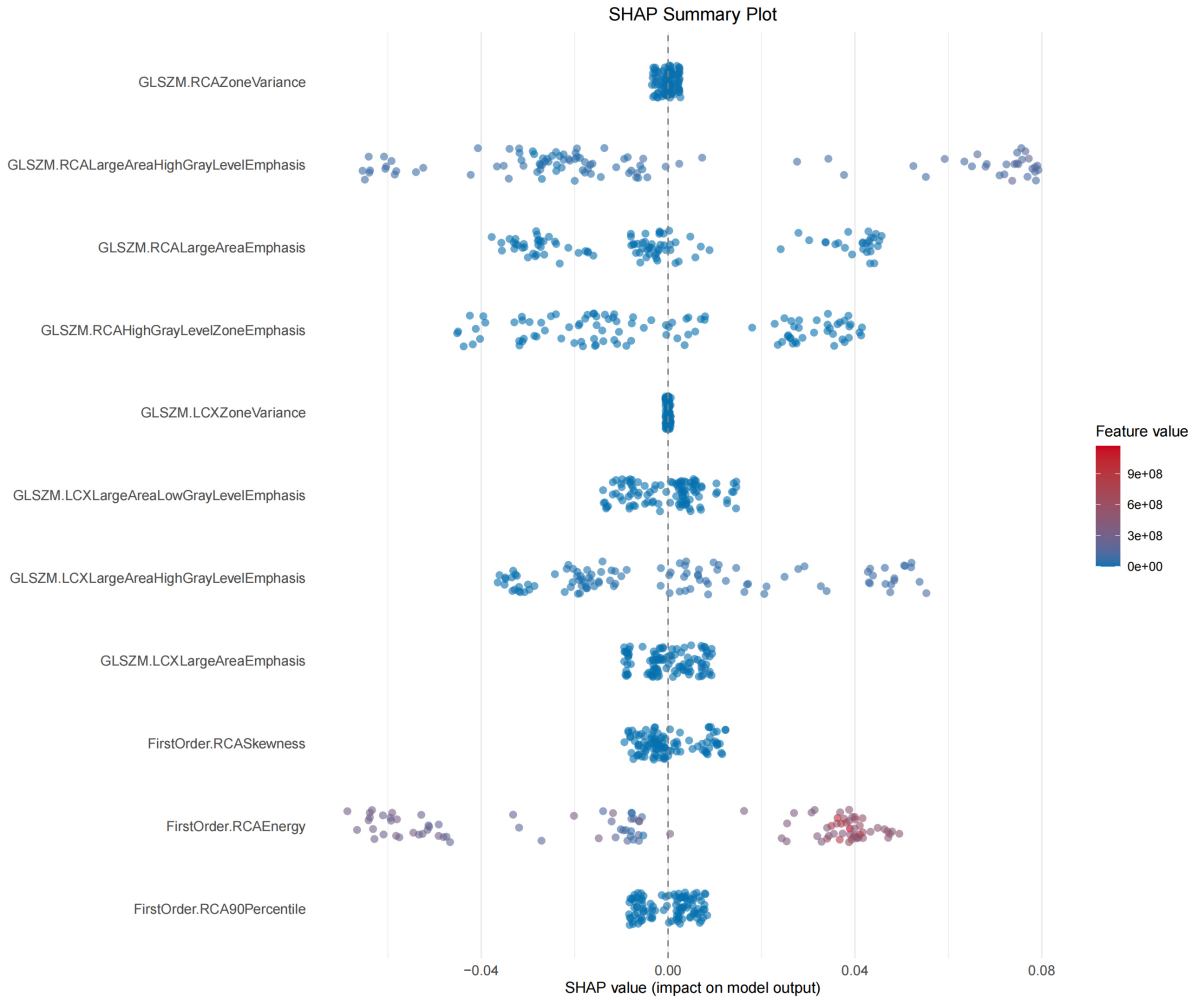
**SHAP summary plot of radiomic features associated with elevated LDL-C levels**. SHAP values indicate the contribution of each radiomic feature to the discrimination of elevated LDL-C levels, with color representing feature values from low (blue) to high (red). Abbreviations: LDL-C, low-density lipoprotein cholesterol; SHAP, shapley additive explanations.

Among various machine learning models, the ensemble GBM model achieved the best performance, with an AUC of 0.889 in the training set and 0.724 in the internal hold-out test set (Fig. 5). SHAP analysis revealed that GLSZM.RCAZoneVariance consistently contributed to the discrimination of elevated LDL-C levels. Notably, the FirstOrder.RCAEnergy showed the highest contribution, whereas large high-gray-level zone features such as GLSZM.RCALargeAreaHighGrayLevelEmphasis and GLSZM.LCXLargeAreaHighGrayLevelEmphasis demonstrated substantial local driving effects for elevated LDL-C levels (Fig. 6). These results suggest that, in this cohort, PCAT regions exhibit more extensive and heterogeneous high-attenuation texture phenotypes in patients with elevated LDL-C levels.

## 4. Discussion

To our knowledge, this study is among the first to evaluate the relationship between elevated LDL-C levels and PCAT phenotypes using CCTA-based radiomics. Individuals with higher lipid exposure demonstrated greater spatial heterogeneity of PCAT, characterized by larger high-attenuation (high-gray-level) regions. These observations are consistent with histologic evidence indicating that inflammation, fibrosis, and neovascularization contribute to adipose tissue inhomogeneity [1, 23, 24]. From a clinical perspective, these imaging changes are consistent with the cumulative metabolic burden of long-term LDL-C elevation, thereby providing imaging-based evidence supporting the link between cholesterol levels and localized coronary inflammation.

We identified several radiomic features associated with elevated LDL-C levels. Notably, second-order GLSZM metrics such as LargeAreaHighGrayLevelEmphasis and ZoneVariance were strongly related to LDL-C status, reflecting clustered regions of high gray-level voxels and increased textual heterogeneity within PCAT. These findings align with histopathological evidence: Mazurek *et al*. [23] demonstrated abundant inflammatory mediators, fibrosis, and microvascular proliferation within epicardial and pericoronary adipose tissue, providing biological support for the imaging manifestations of high-density clustering and increased heterogeneity. Mechanistically, LDL-C may influence PCAT through an “inside-out” signaling pathway, in which endothelial dysfunction activates adipocyte paracrine signaling, thereby promoting immune cell recruitment, lipid droplet remodeling, and stromal reorganization [11, 25, 26]. Animal studies have shown that high-fat diets exacerbate PCAT inflammation and remodeling [27], while human pathological studies corroborate these changes, showing reduced lipid droplet content, fibrosis, and inflammatory cell infiltration in hyperlipidemia and atherosclerosis [28, 29]. Taken together, these findings suggest that chronic LDL-C elevation may promote clustering and heterogeneity in PCAT, reflecting underlying inflammatory and structural remodeling processes, and strengthening the biological plausibility of our results. Nevertheless, these mechanistic interpretations remain hypothesis-generating and warrant independent biological validation in future studies.

Large-scale epidemiological studies have shown that lifelong elevation of LDL-C drives atherosclerosis, with cardiovascular risk increasing proportionally to both concentration and exposure duration [5, 6]. Further evidence indicates that statin therapy is associated with reduced PCAT attenuation, whereas untreated patients do not exhibit such changes [7, 8, 9]. Even after adjustment for potential confounders, changes in LDL-C remain independently correlated with percentage changes in PCAT attenuation. In the present study, first-order radiomic features reflected previously reported attenuation findings, whereas second-order features captured voxel clustering and heterogeneity, thereby providing structural insights beyond mean attenuation alone. These findings are consistent with mechanistic studies by Antonopoulos *et al*. [1], which demonstrated that local coronary inflammation alters PCAT through paracrine signaling, leading to impaired adipocyte maturation, lipid droplet depletion, and increased intracellular water [30, 31]. Collectively, these processes manifest as increased CT attenuation and greater textural complexity on imaging.

In prior work, Antonopoulos *et al*. [1] established PCAT attenuation–based metrics (FAI) as imaging surrogates of coronary inflammation and provided biological rationale linking vascular inflammation to perivascular fat remodeling. In parallel, Lin *et al*. [18] demonstrated that PCAT radiomics can capture spatial patterns beyond voxel intensity alone and may provide incremental discrimination compared with attenuation-only measures in specific clinical settings. Against this background, our study addresses a complementary question by focusing on the patient-level association between LDL-C status and multidimensional PCAT radiomic phenotypes (texture/heterogeneity). Accordingly, the radiomic signatures reported here should be interpreted as exploratory imaging biomarkers that may complement—rather than replace—established attenuation/FAI measures, pending further validation.

Within the framework of residual cardiovascular risk, low-grade inflammation often explains adverse outcomes in statin-treated patients more effectively than residual cholesterol burden [32, 33]. A large percutaneous coronary intervention (PCI) registry showed that high-sensitivity C-reactive protein (hs-CRP) levels ≥2 mg/L, rather than high LDL-C, were independently associated with major adverse cardiovascular events (MACE) [32]. PCAT is recognized as a key imaging window into localized inflammation. Prior studies, including Oikonomou *et al*. [10] and Tzolos *et al*. [13] have supported PCAT attenuation–based metrics (e.g., FAI) as noninvasive markers of coronary inflammation with potential value for risk stratification and associations with adverse cardiovascular outcomes. In this context, we observed that elevated LDL-C levels were associated with increased clustering and heterogeneity within PCAT. These exploratory radiomic signatures may reflect microstructural patterns related to lipid–inflammation interactions and warrant further evaluation alongside established PCAT attenuation/FAI measures in future studies. This perspective not only provides new imaging -based evidence of the LDL-C–PCAT association but also enhances current understanding of lipid–inflammation interactions.

This study extends existing evidence by linking elevated LDL-C levels with PCAT not only through established attenuation patterns but also through novel radiomic signatures of clustering and heterogeneity. These textural features enhance the interpretability of imaging along the lipid–inflammation axis. Importantly, they may serve as external imaging correlates of LDL-C elevation, potentially providing early warning signs of coronary inflammation beyond traditional lipid markers. However, given the single-center retrospective design and the absence of independent external validation, these radiomic signatures should be considered exploratory imaging biomarkers, and their performance and generalizability should be interpreted cautiously. With further validation, these features may evolve into adjunctive tools for therapeutic monitoring and risk stratification. Prospective multicenter studies with independent external validation and outcome follow-up are required to establish generalizability and clinical utility.

## 5. Study Limitations

This single-center, retrospective study with a modest sample size (n = 150) may limit representativeness and generalizability. The machine-learning model was evaluated only internally without independent external validation; therefore, model optimism and potential overfitting cannot be fully excluded and the findings should be interpreted as exploratory. Residual confounding cannot be fully ruled out because multivariable adjustment was not performed, including potential effects of lipid-lowering therapy. In addition, although images were acquired on the same scanner using a standardized protocol, we did not perform formal radiomic reproducibility testing (e.g., ICC/test–retest) and did not assess reproducibility or harmonization across scanners/protocols. Finally, the analysis was cross-sectional and lacked outcome follow-up, precluding assessment of prognostic value and causal inference; the absence of histologic or molecular validation also limits definitive mechanistic interpretation. Future work should include multicenter external validation with longitudinal follow-up and, where feasible, biological validation to establish robustness, generalizability, and clinical utility.

## 6. Conclusions

Elevated LDL-C levels were significantly associated with increased spatial heterogeneity and high gray-level clustering in PCAT, providing imaging-based evidence of the LDL-C–PCAT association. These findings suggest that PCAT radiomic features may serve as external imaging phenotypes of lipid burden, offering more nuanced insight into the relationship between LDL-C and the local coronary microenvironment. Collectively, these radiomic features hold potential clinical value by supporting more precise assessment and management of coronary risk in patients with lipid dysregulation.

## Data Availability

The datasets used in this study are available from the corresponding author upon reasonable request.

## Abbreviations

AUC, area under the curve; CAD, coronary artery disease; CCTA, coronary computed tomography angiography; FAI, fat attenuation index; GBM, gradient boosting machine; GLSZM, gray-level size zone matrix; HDL-C, high-density lipoprotein cholesterol; HU, hounsfield unit; LAD, left anterior descending artery; LCX, left circumflex artery; LDL, low-density lipoprotein; LDL-C, low-density lipoprotein cholesterol; PCAT, pericoronary adipose tissue; RCA, right coronary artery; ROI, region of interest; SHAP, shapley additive explanations; SVM, support vector machine.
