# Supplementary material for: Computed Tomography-Based Radiomics Provides New Insights Into Associations Between Pericoronary Fat Characteristics and Low-Density Lipoprotein Cholesterol
Source: Rev Cardiovasc Med. 2026 May 25;27(5):47037. doi: 10.31083/RCM47037 (PMC13227376; doi:10.31083/RCM47037)
Supplement: Supplementary file 1 [file 2153-8174-27-5-47037-s1.zip › Supplementary Material.docx]

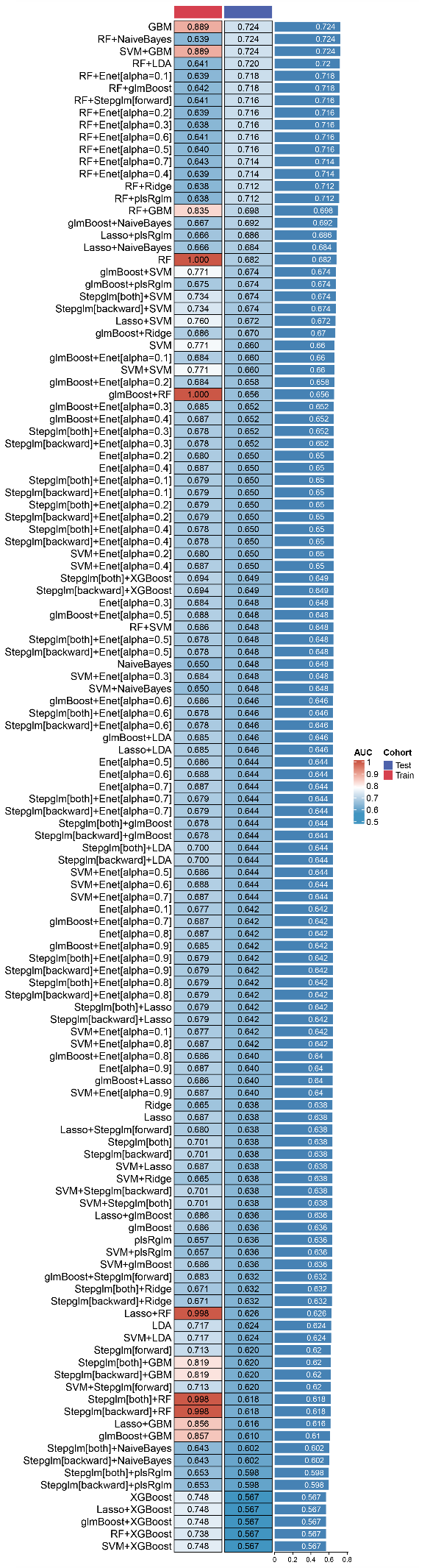


Supplementary Fig. 1

Supplementary Table 1

| **Characteristic** | **1**, N = 76^1^ | **2**, N = 74^1^ | **p-value**^2^ |
| --- | --- | --- | --- |
| RadiomicsFirstOrder.LADMeshVolume.mm3 | 1,946.55 (574.16) | 1,912.03 (552.67) | 0.3 |
| RadiomicsFirstOrder.LADFai.Hu | -76.25 (8.73) | -75.57 (8.60) | 0.3 |
| RadiomicsFirstOrder.LAD10Percentile | -113.58 (12.95) | -112.27 (11.84) | 0.3 |
| RadiomicsFirstOrder.LAD90Percentile | -41.04 (4.10) | -40.86 (4.88) | 0.3 |
| RadiomicsFirstOrder.LADEnergy | 315,497,333.18 (164,870,634.59) | 316,514,476.58 (156,100,954.36) | >0.9 |
| RadiomicsFirstOrder.LADEntropy | 2.13 (0.20) | 2.12 (0.18) | 0.4 |
| RadiomicsFirstOrder.LADInterquartileRange | 39.70 (6.46) | 39.12 (5.61) | 0.4 |
| RadiomicsFirstOrder.LADKurtosis | 3.26 (1.21) | 3.18 (0.88) | 0.3 |
| RadiomicsFirstOrder.LADMaximum |  |  |  |
| -30 | 76 / 76 (100%) | 74 / 74 (100%) |  |
| RadiomicsFirstOrder.LADMeanAbsoluteDeviation | 22.64 (3.25) | 22.22 (2.83) | 0.3 |
| RadiomicsFirstOrderLADMean | -76.30 (8.72) | -75.53 (8.57) | 0.3 |
| RadiomicsFirstOrder.LADMedian | -74.03 (10.25) | -73.24 (9.75) | 0.3 |
| RadiomicsFirstOrder.LADMinimum | -187.45 (8.77) | -187.11 (6.60) | 0.090 |
| RadiomicsFirstOrder.LADRange | 157.45 (8.77) | 157.11 (6.60) | 0.090 |
| RadiomicsFirstOrder.LADRobustMeanAbsoluteDeviation | 16.54 (2.58) | 16.27 (2.26) | 0.4 |
| RadiomicsFirstOrder.LADRootMeanSquared | 81.31 (9.02) | 80.40 (8.83) | 0.3 |
| RadiomicsFirstOrder.LADSkewness | -0.55 (0.41) | -0.55 (0.34) | 0.3 |
| RadiomicsFirstOrder.LADTotalEnergy | 13,620,972.61 (5,873,477.95) | 13,115,235.86 (5,971,320.75) | 0.4 |
| RadiomicsFirstOrder.LADUniformity | 0.26 (0.04) | 0.26 (0.04) | 0.4 |
| RadiomicsFirstOrder.LADVariance | 795.94 (214.27) | 762.36 (185.12) | 0.2 |
| GLCM.LADAutocorrelation | 28.96 (4.13) | 29.23 (4.19) | 0.4 |
| GLCM.LADClusterProminence | 59.01 (31.28) | 54.13 (22.57) | 0.4 |
| GLCM.LADClusterShade | -4.45 (3.68) | -4.25 (2.54) | 0.6 |
| GLCM.LADClusterTendency | 4.31 (1.10) | 4.17 (0.91) | 0.3 |
| GLCM.LADContrast | 0.76 (0.27) | 0.72 (0.24) | 0.094 |
| GLCM.LADCorrelation | 0.69 (0.07) | 0.71 (0.06) | 0.4 |
| GLCM.LADDifferenceAverage | 0.56 (0.11) | 0.54 (0.11) | 0.094 |
| GLCM.LADDifferenceEntropy | 1.29 (0.14) | 1.26 (0.13) | 0.10 |
| GLCM.LADDifferenceVariance | 0.43 (0.13) | 0.40 (0.10) | 0.10 |
| GLCM.LADId | 0.75 (0.04) | 0.76 (0.04) | 0.11 |
| GLCM.LADIdm | 0.74 (0.04) | 0.75 (0.04) | 0.11 |
| GLCM.LADIdmn | 0.99 (0.00) | 0.99 (0.00) | 0.12 |
| GLCM.LADIdn | 0.93 (0.01) | 0.93 (0.01) | 0.10 |
| GLCM.LADImc1 | -0.25 (0.05) | -0.26 (0.05) | 0.093 |
| GLCM.LADImc2 | 0.78 (0.07) | 0.79 (0.06) | 0.4 |
| GLCM.LADInverseVariance | 0.42 (0.03) | 0.42 (0.03) | 0.2 |
| GLCM.LADJointAverage | 5.28 (0.40) | 5.31 (0.41) | 0.4 |
| GLCM.LADJointEnergy | 0.11 (0.03) | 0.11 (0.03) | 0.3 |
| GLCM.LADJointEntropy | 3.72 (0.35) | 3.68 (0.32) | 0.3 |
| GLCM.LADMCC | 0.71 (0.06) | 0.72 (0.06) | 0.2 |
| GLCM.LADMaximumProbability | 0.19 (0.05) | 0.19 (0.05) | 0.5 |
| GLCM.LADSumAverage | 10.57 (0.81) | 10.62 (0.81) | 0.4 |
| GLCM.LADSumEntropy | 2.95 (0.22) | 2.94 (0.19) | 0.4 |
| GLCM.LADSumSquares | 1.27 (0.32) | 1.22 (0.27) | 0.2 |
| GLSZM.LADGrayLevelNonUniformity | 62.36 (28.11) | 60.80 (23.60) | 0.8 |
| GLSZM.LADGrayLevelNonUniformityNormalized | 0.18 (0.03) | 0.19 (0.04) | 0.5 |
| GLSZM.LADGrayLevelVariance | 3.51 (0.74) | 3.40 (0.79) | 0.4 |
| GLSZM.LADHighGrayLevelZoneEmphasis | 22.91 (4.22) | 23.71 (4.55) | 0.5 |
| GLSZM.LADLargeAreaEmphasis | 1,577,568.85 (1,093,771.53) | 1,881,780.55 (1,311,351.40) | 0.2 |
| GLSZM.LADLargeAreaHighGrayLevelEmphasis | 49,831,702.80 (33,322,981.27) | 60,497,691.43 (40,674,017.77) | 0.2 |
| GLSZM.LADLargeAreaLowGrayLevelEmphasis | 56,190.27 (43,560.34) | 66,019.09 (54,710.93) | 0.4 |
| GLSZM.LADLowGrayLevelZoneEmphasis | 0.15 (0.06) | 0.14 (0.06) | 0.2 |
| GLSZM.LADSizeZoneNonUniformity | 63.90 (53.16) | 64.66 (54.33) | 0.6 |
| GLSZM.LADSizeZoneNonUniformityNormalized | 0.16 (0.06) | 0.16 (0.06) | 0.7 |
| GLSZM.LADSmallAreaEmphasis | 0.39 (0.08) | 0.39 (0.09) | 0.7 |
| GLSZM.LADSmallAreaHighGrayLevelEmphasis | 9.57 (2.22) | 9.92 (1.95) | 0.4 |
| GLSZM.LADSmallAreaLowGrayLevelEmphasis | 0.07 (0.05) | 0.07 (0.05) | 0.4 |
| GLSZM.LADZoneEntropy | 5.79 (0.25) | 5.76 (0.22) | 0.3 |
| GLSZM.LADZonePercentage | 0.01 (0.01) | 0.01 (0.01) | 0.2 |
| GLSZM.LADZoneVariance | 1,550,320.09 (1,073,343.19) | 1,846,599.85 (1,285,475.56) | 0.2 |
| GLRLM.LADGrayLevelNonUniformity | 5,929.25 (1,963.48) | 6,101.67 (1,619.96) | 0.6 |
| GLRLM.LADGrayLevelNonUniformityNormalized | 0.25 (0.04) | 0.25 (0.04) | 0.3 |
| GLRLM.LADGrayLevelVariance | 1.48 (0.36) | 1.42 (0.32) | 0.2 |
| GLRLM.LADHighGrayLevelRunEmphasis | 31.18 (3.90) | 31.44 (4.09) | 0.5 |
| GLRLM.LADLongRunEmphasis | 5.86 (1.53) | 6.20 (1.62) | 0.11 |
| GLRLM.LADLongRunHighGrayLevelEmphasis | 173.70 (50.15) | 185.59 (51.97) | 0.11 |
| GLRLM.LADLongRunLowGrayLevelEmphasis | 0.25 (0.08) | 0.26 (0.09) | 0.7 |
| GLRLM.LADLowGrayLevelRunEmphasis | 0.05 (0.01) | 0.05 (0.01) | 0.4 |
| GLRLM.LADRunEntropy | 3.95 (0.25) | 3.97 (0.25) | 0.6 |
| GLRLM.LADRunLengthNonUniformity | 9,978.79 (4,339.29) | 9,812.76 (2,468.56) | 0.9 |
| GLRLM.LADRunLengthNonUniformityNormalized | 0.40 (0.06) | 0.39 (0.06) | 0.15 |
| GLRLM.LADRunPercentage | 0.55 (0.06) | 0.54 (0.06) | 0.2 |
| GLRLM.LADRunVariance | 2.09 (0.70) | 2.25 (0.75) | 0.11 |
| GLRLM.LADShortRunEmphasis | 0.64 (0.05) | 0.63 (0.06) | 0.13 |
| GLRLM.LADShortRunHighGrayLevelEmphasis | 20.50 (2.82) | 20.42 (3.03) | 0.7 |
| GLRLM.LADShortRunLowGrayLevelEmphasis | 0.03 (0.01) | 0.03 (0.01) | 0.3 |
| NGTDM.LADBusyness | 111.34 (51.53) | 110.32 (37.98) | >0.9 |
| NGTDM.LADCoarseness | 0.00 (0.00) | 0.00 (0.00) | 0.6 |
| NGTDM.LADComplexity | 9.83 (1.86) | 9.69 (1.85) | 0.7 |
| NGTDM.LADContrast | 0.03 (0.01) | 0.03 (0.01) | 0.2 |
| NGTDM.LADStrength | 0.01 (0.01) | 0.01 (0.01) | >0.9 |
| GLDM.LADDependenceEntropy | 6.45 (0.23) | 6.46 (0.21) | 0.9 |
| GLDM.LADDependenceNonUniformity | 2,332.79 (939.50) | 2,366.75 (700.44) | 0.8 |
| GLDM.LADDependenceNonUniformityNormalized | 0.05 (0.01) | 0.05 (0.01) | 0.4 |
| GLDM.LADDependenceVariance | 28.61 (5.05) | 29.08 (5.12) | 0.4 |
| GLDM.LADGrayLevelNonUniformity | 11,391.50 (4,266.06) | 11,971.22 (4,261.42) | 0.5 |
| GLDM.LADGrayLevelVariance | 1.34 (0.34) | 1.28 (0.30) | 0.2 |
| GLDM.LADHighGrayLevelEmphasis | 30.50 (4.20) | 30.76 (4.32) | 0.5 |
| GLDM.LADLargeDependenceEmphasis | 190.02 (41.84) | 196.86 (43.80) | 0.2 |
| GLDM.LADLargeDependenceHighGrayLevelEmphasis | 5,531.26 (1,413.85) | 5,790.85 (1,471.41) | 0.2 |
| GLDM.LADLargeDependenceLowGrayLevelEmphasis | 8.23 (2.61) | 8.36 (2.76) | >0.9 |
| GLDM.LADLowGrayLevelEmphasis | 0.05 (0.01) | 0.04 (0.01) | 0.3 |
| GLDM.LADSmallDependenceEmphasis | 0.02 (0.01) | 0.02 (0.01) | 0.2 |
| GLDM.LADSmallDependenceHighGrayLevelEmphasis | 0.58 (0.28) | 0.55 (0.21) | 0.4 |
| GLDM.LADSmallDependenceLowGrayLevelEmphasis | 0.00 (0.00) | 0.00 (0.00) | 0.2 |
| FirstOrder.LCXLength.mm |  |  |  |
| 0-40 | 76 / 76 (100%) | 74 / 74 (100%) |  |
| FirstOrder.LCXMeshVolume.mm3 | 1,363.99 (563.90) | 1,444.41 (450.99) | 0.6 |
| FirstOrder.LCXFai.Hu | -73.84 (9.98) | -74.35 (7.37) | 0.8 |
| FirstOrder.LCX10Percentile | -113.13 (16.37) | -113.97 (12.53) | >0.9 |
| FirstOrder.LCX90Percentile | -38.92 (3.57) | -39.11 (3.45) | 0.9 |
| FirstOrder.LCXEnergy | 215,964,737.42 (133,718,276.17) | 235,577,244.36 (123,571,406.36) | 0.3 |
| FirstOrder.LCXEntropy | 2.13 (0.28) | 2.15 (0.19) | >0.9 |
| FirstOrder.LCXInterquartileRange | 40.84 (7.75) | 41.16 (6.41) | >0.9 |
| FirstOrder.LCXKurtosis | 3.21 (1.07) | 3.19 (0.78) | 0.8 |
| FirstOrder.LCXMaximum |  |  |  |
| -30 | 76 / 76 (100%) | 74 / 74 (100%) |  |
| FirstOrder.LCXMeanAbsoluteDeviation | 23.16 (4.09) | 23.36 (3.34) | >0.9 |
| FirstOrder.LCXMean | -73.89 (9.93) | -74.36 (7.38) | 0.9 |
| FirstOrder.LCXMedian | -70.49 (10.59) | -70.89 (7.97) | >0.9 |
| FirstOrder.LCXMinimum | -182.78 (17.19) | -186.32 (8.30) | 0.5 |
| FirstOrder.LCXRange | 152.78 (17.19) | 156.32 (8.30) | 0.5 |
| FirstOrder.LCXRobustMeanAbsoluteDeviation | 16.97 (3.15) | 17.12 (2.58) | >0.9 |
| FirstOrder.LCXRootMeanSquared | 79.24 (10.79) | 79.77 (7.99) | 0.8 |
| FirstOrder.LCXSkewness | -0.66 (0.33) | -0.66 (0.26) | 0.8 |
| FirstOrder.LCXTotalEnergy | 9,427,722.93 (5,384,992.76) | 9,627,977.71 (4,282,758.72) | 0.8 |
| FirstOrder.LCXUniformity | 0.26 (0.06) | 0.25 (0.04) | >0.9 |
| FirstOrder.LCXVariance | 837.40 (268.52) | 843.06 (229.39) | 0.9 |
| GLCM.LCXAutocorrelation | 27.95 (5.31) | 29.23 (3.21) | 0.2 |
| GLCM.LCXClusterProminence | 66.87 (34.47) | 68.64 (32.36) | 0.9 |
| GLCM.LCXClusterShade | -5.72 (2.97) | -6.10 (3.11) | 0.5 |
| GLCM.LCXClusterTendency | 4.54 (1.41) | 4.62 (1.20) | >0.9 |
| GLCM.LCXContrast | 0.88 (0.29) | 0.86 (0.32) | 0.3 |
| GLCM.LCXCorrelation | 0.67 (0.07) | 0.69 (0.06) | 0.089 |
| GLCM.LCXDifferenceAverage | 0.62 (0.12) | 0.61 (0.13) | 0.3 |
| GLCM.LCXDifferenceEntropy | 1.36 (0.15) | 1.35 (0.15) | 0.3 |
| GLCM.LCXDifferenceVariance | 0.46 (0.12) | 0.45 (0.12) | 0.2 |
| GLCM.LCXId | 0.73 (0.04) | 0.73 (0.04) | 0.3 |
| GLCM.LCXIdm | 0.71 (0.05) | 0.72 (0.05) | 0.2 |
| GLCM.LCXIdmn | 0.98 (0.01) | 0.98 (0.01) | 0.014 |
| GLCM.LCXIdn | 0.92 (0.01) | 0.93 (0.01) | 0.026 |
| GLCM.LCXImc1 | -0.22 (0.04) | -0.23 (0.05) | 0.021 |
| GLCM.LCXImc2 | 0.75 (0.08) | 0.77 (0.06) | 0.13 |
| GLCM.LCXInverseVariance | 0.44 (0.03) | 0.44 (0.03) | 0.2 |
| GLCM.LCXJointAverage | 5.17 (0.55) | 5.31 (0.31) | 0.2 |
| GLCM.LCXJointEnergy | 0.10 (0.04) | 0.10 (0.02) | 0.7 |
| GLCM.LCXJointEntropy | 3.83 (0.45) | 3.85 (0.35) | 0.7 |
| GLCM.LCXMCC | 0.69 (0.08) | 0.71 (0.06) | 0.10 |
| GLCM.LCXMaximumProbability | 0.17 (0.06) | 0.17 (0.04) | 0.6 |
| GLCM.LCXSumAverage | 10.34 (1.11) | 10.62 (0.62) | 0.2 |
| GLCM.LCXSumEntropy | 2.97 (0.29) | 3.00 (0.20) | >0.9 |
| GLCM.LCXSumSquares | 1.35 (0.40) | 1.37 (0.35) | >0.9 |
| GLSZM.LCXGrayLevelNonUniformity | 47.68 (23.05) | 46.27 (16.65) | 0.8 |
| GLSZM.LCXGrayLevelNonUniformityNormalized | 0.20 (0.04) | 0.19 (0.03) | 0.11 |
| GLSZM.LCXGrayLevelVariance | 3.23 (1.01) | 3.31 (0.84) | >0.9 |
| GLSZM.LCXHighGrayLevelZoneEmphasis | 24.31 (5.32) | 24.89 (3.86) | >0.9 |
| GLSZM.LCXLargeAreaEmphasis | 941,498.25 (724,021.60) | 1,233,463.49 (913,611.61) | 0.060 |
| GLSZM.LCXLargeAreaHighGrayLevelEmphasis | 31,203,146.73 (23,129,132.79) | 42,010,724.41 (28,273,527.18) | 0.022 |
| GLSZM.LCXLargeAreaLowGrayLevelEmphasis | 31,714.89 (26,004.99) | 40,107.96 (35,302.97) | 0.2 |
| GLSZM.LCXLowGrayLevelZoneEmphasis | 0.12 (0.05) | 0.12 (0.05) | 0.5 |
| GLSZM.LCXSizeZoneNonUniformity | 35.53 (33.74) | 33.77 (26.30) | 0.8 |
| GLSZM.LCXSizeZoneNonUniformityNormalized | 0.12 (0.05) | 0.12 (0.04) | 0.6 |
| GLSZM.LCXSmallAreaEmphasis | 0.33 (0.08) | 0.34 (0.07) | 0.6 |
| GLSZM.LCXSmallAreaHighGrayLevelEmphasis | 9.68 (2.85) | 10.01 (2.40) | >0.9 |
| GLSZM.LCXSmallAreaLowGrayLevelEmphasis | 0.04 (0.04) | 0.04 (0.04) | 0.7 |
| GLSZM.LCXZoneEntropy | 5.84 (0.30) | 5.91 (0.24) | 0.2 |
| GLSZM.LCXZonePercentage | 0.01 (0.01) | 0.01 (0.01) | 0.035 |
| GLSZM.LCXZoneVariance | 919,117.74 (709,033.63) | 1,203,602.70 (893,656.37) | 0.065 |
| GLRLM.LCXGrayLevelNonUniformity | 4,488.14 (1,926.33) | 4,983.40 (1,551.25) | 0.083 |
| GLRLM.LCXGrayLevelNonUniformityNormalized | 0.26 (0.06) | 0.25 (0.04) | >0.9 |
| GLRLM.LCXGrayLevelVariance | 1.50 (0.46) | 1.51 (0.37) | 0.8 |
| GLRLM.LCXHighGrayLevelRunEmphasis | 30.43 (5.44) | 31.80 (3.15) | 0.3 |
| GLRLM.LCXLongRunEmphasis | 4.54 (1.08) | 4.83 (1.20) | 0.056 |
| GLRLM.LCXLongRunHighGrayLevelEmphasis | 133.42 (42.04) | 147.40 (37.15) | 0.008 |
| GLRLM.LCXLongRunLowGrayLevelEmphasis | 0.21 (0.08) | 0.21 (0.06) | 0.7 |
| GLRLM.LCXLowGrayLevelRunEmphasis | 0.05 (0.02) | 0.05 (0.01) | 0.10 |
| GLRLM.LCXRunEntropy | 3.75 (0.29) | 3.81 (0.25) | 0.2 |
| GLRLM.LCXRunLengthNonUniformity | 8,475.75 (4,317.48) | 8,967.10 (2,861.59) | 0.3 |
| GLRLM.LCXRunLengthNonUniformityNormalized | 0.45 (0.06) | 0.44 (0.07) | 0.056 |
| GLRLM.LCXRunPercentage | 0.60 (0.05) | 0.59 (0.06) | 0.054 |
| GLRLM.LCXRunVariance | 1.49 (0.50) | 1.62 (0.54) | 0.048 |
| GLRLM.LCXShortRunEmphasis | 0.69 (0.05) | 0.68 (0.05) | 0.068 |
| GLRLM.LCXShortRunHighGrayLevelEmphasis | 21.26 (3.98) | 21.92 (2.47) | 0.4 |
| GLRLM.LCXShortRunLowGrayLevelEmphasis | 0.04 (0.01) | 0.03 (0.01) | 0.042 |
| NGTDM.LCXBusyness | 84.56 (39.97) | 88.84 (34.21) | 0.5 |
| NGTDM.LCXCoarseness | 0.00 (0.00) | 0.00 (0.00) | 0.10 |
| NGTDM.LCXComplexity | 10.28 (2.50) | 10.57 (1.83) | >0.9 |
| NGTDM.LCXContrast | 0.03 (0.01) | 0.03 (0.01) | 0.2 |
| NGTDM.LCXStrength | 0.01 (0.01) | 0.01 (0.01) | 0.5 |
| GLDM.LCXDependenceEntropy | 6.33 (0.29) | 6.39 (0.24) | 0.4 |
| GLDM.LCXDependenceNonUniformity | 1,796.76 (883.69) | 1,965.92 (648.69) | 0.2 |
| GLDM.LCXDependenceNonUniformityNormalized | 0.06 (0.01) | 0.06 (0.01) | 0.082 |
| GLDM.LCXDependenceVariance | 24.47 (5.17) | 25.23 (5.36) | 0.094 |
| GLDM.LCXGrayLevelNonUniformity | 7,749.72 (3,545.45) | 8,833.55 (3,347.56) | 0.063 |
| GLDM.LCXGrayLevelVariance | 1.40 (0.44) | 1.41 (0.37) | 0.9 |
| GLDM.LCXHighGrayLevelEmphasis | 29.89 (5.49) | 31.18 (3.25) | 0.3 |
| GLDM.LCXLargeDependenceEmphasis | 155.18 (36.17) | 163.56 (40.42) | 0.049 |
| GLDM.LCXLargeDependenceHighGrayLevelEmphasis | 4,455.02 (1,413.19) | 4,880.96 (1,290.82) | 0.012 |
| GLDM.LCXLargeDependenceLowGrayLevelEmphasis | 7.22 (2.54) | 7.01 (2.12) | 0.8 |
| GLDM.LCXLowGrayLevelEmphasis | 0.05 (0.02) | 0.04 (0.01) | 0.12 |
| GLDM.LCXSmallDependenceEmphasis | 0.02 (0.01) | 0.02 (0.01) | 0.029 |
| GLDM.LCXSmallDependenceHighGrayLevelEmphasis | 0.65 (0.26) | 0.65 (0.28) | 0.3 |
| GLDM.LCXSmallDependenceLowGrayLevelEmphasis | 0.00 (0.00) | 0.00 (0.00) | 0.13 |
| FirstOrder.RCALength.mm |  |  |  |
| 10-50 | 76 / 76 (100%) | 74 / 74 (100%) |  |
| FirstOrder.RCAMeshVolume.mm3 | 2,062.64 (612.96) | 2,269.84 (556.68) | 0.060 |
| FirstOrder.RCAFai.Hu | -77.47 (9.23) | -79.45 (9.06) | 0.2 |
| FirstOrder.RCA10Percentile | -115.41 (14.10) | -117.04 (12.48) | 0.8 |
| FirstOrder.RCA90Percentile | -41.78 (4.48) | -43.28 (5.58) | 0.13 |
| FirstOrder.RCAEnergy | 345,808,452.99 (174,913,746.70) | 419,097,810.50 (199,166,271.23) | 0.005 |
| FirstOrder.RCAEntropy | 2.16 (0.22) | 2.17 (0.19) | 0.6 |
| FirstOrder.RCAInterquartileRange | 39.97 (6.33) | 39.88 (5.57) | 0.5 |
| FirstOrder.RCAKurtosis | 3.20 (1.19) | 2.97 (0.64) | 0.2 |
| FirstOrder.RCAMaximum |  |  |  |
| -30 | 76 / 76 (100%) | 74 / 74 (100%) |  |
| FirstOrder.RCAMeanAbsoluteDeviation | 22.87 (3.39) | 22.80 (2.99) | 0.5 |
| FirstOrder.RCAMean | -77.49 (9.28) | -79.43 (9.05) | 0.3 |
| FirstOrder.RCAMedian | -75.11 (10.35) | -77.58 (10.49) | 0.2 |
| FirstOrder.RCAMinimum | -184.75 (11.74) | -186.08 (8.18) | 0.12 |
| FirstOrder.RCARange | 154.75 (11.74) | 156.08 (8.18) | 0.12 |
| FirstOrder.RCARobustMeanAbsoluteDeviation | 16.64 (2.59) | 16.62 (2.28) | 0.5 |
| FirstOrder.RCARootMeanSquared | 82.53 (9.76) | 84.31 (9.28) | 0.3 |
| FirstOrder.RCASkewness | -0.55 (0.37) | -0.43 (0.33) | 0.054 |
| FirstOrder.RCATotalEnergy | 14,983,527.90 (6,268,627.59) | 16,894,472.91 (6,118,443.28) | 0.044 |
| FirstOrder.RCAUniformity | 0.25 (0.05) | 0.25 (0.04) | 0.6 |
| FirstOrder.RCAVariance | 816.02 (224.25) | 802.69 (209.73) | 0.4 |
| GLCM.RCAAutocorrelation | 27.43 (4.55) | 27.48 (3.67) | 0.9 |
| GLCM.RCAClusterProminence | 63.21 (32.44) | 60.85 (30.02) | 0.5 |
| GLCM.RCAClusterShade | -4.73 (3.03) | -4.11 (2.99) | 0.2 |
| GLCM.RCAClusterTendency | 4.48 (1.27) | 4.48 (1.11) | 0.8 |
| GLCM.RCAContrast | 0.73 (0.20) | 0.69 (0.21) | 0.064 |
| GLCM.RCACorrelation | 0.71 (0.07) | 0.73 (0.06) | 0.040 |
| GLCM.RCADifferenceAverage | 0.55 (0.10) | 0.53 (0.10) | 0.12 |
| GLCM.RCADifferenceEntropy | 1.28 (0.12) | 1.25 (0.13) | 0.089 |
| GLCM.RCADifferenceVariance | 0.41 (0.09) | 0.39 (0.09) | 0.040 |
| GLCM.RCAId | 0.75 (0.03) | 0.76 (0.04) | 0.13 |
| GLCM.RCAIdm | 0.74 (0.04) | 0.75 (0.04) | 0.12 |
| GLCM.RCAIdmn | 0.99 (0.00) | 0.99 (0.00) | 0.006 |
| GLCM.RCAIdn | 0.93 (0.01) | 0.93 (0.01) | 0.023 |
| GLCM.RCAImc1 | -0.26 (0.05) | -0.27 (0.05) | 0.019 |
| GLCM.RCAImc2 | 0.80 (0.08) | 0.81 (0.07) | 0.069 |
| GLCM.RCAInverseVariance | 0.42 (0.04) | 0.42 (0.03) | 0.2 |
| GLCM.RCAJointAverage | 5.13 (0.46) | 5.14 (0.37) | 0.9 |
| GLCM.RCAJointEnergy | 0.10 (0.03) | 0.10 (0.02) | 0.4 |
| GLCM.RCAJointEntropy | 3.74 (0.35) | 3.73 (0.30) | 0.4 |
| GLCM.RCAMCC | 0.73 (0.07) | 0.74 (0.06) | 0.075 |
| GLCM.RCAMaximumProbability | 0.19 (0.05) | 0.19 (0.04) | 0.3 |
| GLCM.RCASumAverage | 10.25 (0.91) | 10.28 (0.73) | 0.9 |
| GLCM.RCASumEntropy | 2.99 (0.24) | 3.00 (0.19) | 0.9 |
| GLCM.RCASumSquares | 1.30 (0.34) | 1.29 (0.31) | 0.6 |
| GLSZM.RCAGrayLevelNonUniformity | 58.11 (30.02) | 56.08 (22.75) | 0.6 |
| GLSZM.RCAGrayLevelNonUniformityNormalized | 0.19 (0.03) | 0.20 (0.03) | 0.047 |
| GLSZM.RCAGrayLevelVariance | 3.46 (0.87) | 3.45 (0.84) | 0.7 |
| GLSZM.RCAHighGrayLevelZoneEmphasis | 21.74 (4.60) | 23.30 (4.07) | 0.049 |
| GLSZM.RCALargeAreaEmphasis | 1,972,960.51 (1,576,641.59) | 2,822,668.84 (1,899,667.77) | 0.002 |
| GLSZM.RCALargeAreaHighGrayLevelEmphasis | 57,547,703.56 (40,270,849.35) | 82,603,165.75 (49,443,918.82) | <0.001 |
| GLSZM.RCALargeAreaLowGrayLevelEmphasis | 78,381.81 (83,936.84) | 109,080.80 (91,499.77) | 0.005 |
| GLSZM.RCALowGrayLevelZoneEmphasis | 0.16 (0.06) | 0.14 (0.07) | 0.008 |
| GLSZM.RCASizeZoneNonUniformity | 54.65 (50.42) | 42.93 (39.40) | 0.12 |
| GLSZM.RCASizeZoneNonUniformityNormalized | 0.15 (0.06) | 0.13 (0.04) | 0.023 |
| GLSZM.RCASmallAreaEmphasis | 0.37 (0.09) | 0.34 (0.07) | 0.027 |
| GLSZM.RCASmallAreaHighGrayLevelEmphasis | 9.04 (2.75) | 9.22 (2.07) | 0.7 |
| GLSZM.RCASmallAreaLowGrayLevelEmphasis | 0.06 (0.05) | 0.05 (0.04) | 0.009 |
| GLSZM.RCAZoneEntropy | 5.87 (0.29) | 5.95 (0.23) | 0.2 |
| GLSZM.RCAZonePercentage | 0.01 (0.01) | 0.01 (0.01) | 0.005 |
| GLSZM.RCAZoneVariance | 1,935,405.79 (1,543,258.77) | 2,770,715.05 (1,867,492.05) | 0.002 |
| GLRLM.RCAGrayLevelNonUniformity | 6,051.42 (2,098.77) | 6,773.27 (1,690.71) | 0.011 |
| GLRLM.RCAGrayLevelNonUniformityNormalized | 0.24 (0.05) | 0.24 (0.04) | 0.8 |
| GLRLM.RCAGrayLevelVariance | 1.52 (0.38) | 1.49 (0.37) | 0.3 |
| GLRLM.RCAHighGrayLevelRunEmphasis | 29.35 (4.52) | 29.55 (3.65) | >0.9 |
| GLRLM.RCALongRunEmphasis | 5.71 (1.50) | 6.19 (1.74) | 0.034 |
| GLRLM.RCALongRunHighGrayLevelEmphasis | 159.37 (41.87) | 171.98 (42.27) | 0.024 |
| GLRLM.RCALongRunLowGrayLevelEmphasis | 0.27 (0.11) | 0.29 (0.11) | 0.2 |
| GLRLM.RCALowGrayLevelRunEmphasis | 0.05 (0.01) | 0.05 (0.01) | 0.2 |
| GLRLM.RCARunEntropy | 4.00 (0.29) | 4.07 (0.31) | 0.043 |
| GLRLM.RCARunLengthNonUniformity | 10,254.95 (4,606.86) | 10,946.50 (2,763.21) | 0.040 |
| GLRLM.RCARunLengthNonUniformityNormalized | 0.39 (0.06) | 0.38 (0.07) | 0.028 |
| GLRLM.RCARunPercentage | 0.55 (0.06) | 0.53 (0.07) | 0.035 |
| GLRLM.RCARunVariance | 2.00 (0.68) | 2.21 (0.79) | 0.041 |
| GLRLM.RCAShortRunEmphasis | 0.64 (0.05) | 0.62 (0.06) | 0.027 |
| GLRLM.RCAShortRunHighGrayLevelEmphasis | 19.18 (3.52) | 18.95 (3.08) | 0.3 |
| GLRLM.RCAShortRunLowGrayLevelEmphasis | 0.03 (0.01) | 0.03 (0.01) | 0.011 |
| NGTDM.RCABusyness | 122.75 (62.60) | 131.58 (43.59) | 0.017 |
| NGTDM.RCACoarseness | 0.00 (0.00) | 0.00 (0.00) | 0.009 |
| NGTDM.RCAComplexity | 9.03 (2.13) | 8.86 (1.84) | 0.2 |
| NGTDM.RCAContrast | 0.03 (0.01) | 0.03 (0.01) | 0.050 |
| NGTDM.RCAStrength | 0.01 (0.02) | 0.01 (0.01) | 0.022 |
| GLDM.RCADependenceEntropy | 6.47 (0.27) | 6.49 (0.27) | >0.9 |
| GLDM.RCADependenceNonUniformity | 2,513.48 (1,022.23) | 2,872.58 (802.99) | 0.003 |
| GLDM.RCADependenceNonUniformityNormalized | 0.05 (0.01) | 0.05 (0.01) | 0.3 |
| GLDM.RCADependenceVariance | 27.79 (5.16) | 28.08 (5.50) | 0.4 |
| GLDM.RCAGrayLevelNonUniformity | 11,804.40 (4,617.00) | 13,782.51 (4,805.60) | 0.006 |
| GLDM.RCAGrayLevelVariance | 1.37 (0.36) | 1.35 (0.34) | 0.4 |
| GLDM.RCAHighGrayLevelEmphasis | 28.83 (4.71) | 28.86 (3.88) | 0.8 |
| GLDM.RCALargeDependenceEmphasis | 192.56 (42.81) | 203.68 (47.45) | 0.045 |
| GLDM.RCALargeDependenceHighGrayLevelEmphasis | 5,295.19 (1,290.54) | 5,574.86 (1,247.04) | 0.069 |
| GLDM.RCALargeDependenceLowGrayLevelEmphasis | 9.18 (3.56) | 9.54 (3.30) | 0.2 |
| GLDM.RCALowGrayLevelEmphasis | 0.05 (0.01) | 0.05 (0.01) | 0.4 |
| GLDM.RCASmallDependenceEmphasis | 0.02 (0.01) | 0.02 (0.01) | 0.011 |
| GLDM.RCASmallDependenceHighGrayLevelEmphasis | 0.51 (0.23) | 0.46 (0.18) | 0.045 |
| GLDM.RCASmallDependenceLowGrayLevelEmphasis | 0.00 (0.00) | 0.00 (0.00) | 0.001 |
| ^1^Mean (SD); n / N (%) | | | |
| ^2^Wilcoxon rank sum test | | | |
